# The association between financial strain, psychological distress and subsequent depression: findings from a Norwegian national study

**DOI:** 10.1192/bjo.2025.10878

**Published:** 2025-10-24

**Authors:** Børge Sivertsen, Mari Hysing, Tormod Bøe

**Affiliations:** Department of Health Promotion, Norwegian Institute of Public Health, Bergen, Norway; Department of Research and Innovation, Helse Fonna HF, Haugesund, Norway; Department of Psychosocial Science, Faculty of Psychology, University of Bergen, Bergen, Norway

**Keywords:** Financial strain, depression, higher education students, mental health, longitudinal study

## Abstract

**Background:**

Financial strain is increasingly recognised as a contributor to psychological distress, which may in turn elevate the risk of developing mental disorder. However, few large-scale longitudinal studies have investigated its predictive role using diagnostic outcomes among higher education students.

**Aims:**

To examine whether financial strain predicts a major depressive episode (MDE) one year later among Norwegian students, and whether associations are explained by sociodemographic factors or baseline psychological distress.

**Method:**

Data were drawn from the national Students’ Health and Wellbeing Study 2022 (SHoT2022) survey (*N* = 53 362), with a diagnostic follow-up one year later (*N* = 10 460) using the self-administered Composite International Diagnostic Interview version 5.0 (CIDI 5.0). Inverse probability weighted Poisson regression with robust standard errors estimated the risk of 30-day DSM-5-defined MDE for each financial indicator.

**Results:**

Financial strain was widespread: 6% reported frequent financial difficulties, 27% were unable to cover an emergency expense of 5000 Norwegian kroner (NOK; approximately €450/$500, and 35% spent 60% or more of their income on housing. Several indicators significantly predicted later MDE. Students frequently experiencing financial difficulties had a 3.55-fold increased risk (95% CI:2.97–4.22), attenuating to 1.53 (1.28–1.83) after full adjustment. Similar patterns emerged for most indicators. Associations were largely unaffected by sociodemographic adjustment, but were substantially reduced after accounting for baseline psychological distress.

**Conclusions:**

Financial strain was associated with increased risk of MDE one year later, although much of the association was explained by baseline distress. Policies should address both financial and psychological vulnerabilities through strengthened financial support, alignment with living costs and targeted measures such as financial counselling and housing assistance.

The transition to higher education marks a critical developmental phase, accompanied by increased academic pressure, social changes and financial responsibilities. While higher education has traditionally been associated with long-term socioeconomic and health benefits,^1,2^ a growing body of evidence suggests that students experience disproportionately high rates of mental health problems compared to non-student peers.^3,4^ Depression, anxiety and suicidal ideation are now consistently reported at elevated levels among university populations globally, including in high-income countries where access to education is relatively equitable.^5–7^ However, equitable access does not eliminate challenges related to living expenses, nor broader psychological and financial barriers such as fear of debt or poverty of aspiration.^8^

Among potential risk factors for mental health problems, financial stress has received increasing attention. Studies have shown that both objective financial strain (e.g. low income, debt or difficulties covering expenses) and subjective perceptions of financial insecurity are associated with adverse mental health outcomes among students.^9–11^ Financial difficulties have also been linked to self-harm, suicidal behaviour, and academic underperformance and non-continuation.^12,13^ Importantly, these patterns persist even in contexts like Norway, where higher education is largely publicly funded and student support is comparatively generous.^14^ Despite this support, many Norwegian students struggle to meet basic living costs and accumulate substantial debt during their studies.

Research based on the SHoT (Students’ Health and Wellbeing Study) survey from 2018 has highlighted a robust cross-sectional association between financial difficulties and multiple dimensions of student health.^14^ However, much of the current literature is limited by the use of self-reported mental health outcomes, cross-sectional designs or single-institution samples.^15^ Moreover, the economic downturn following the COVID-19 pandemic, characterised by rising inflation and surging energy costs,^16^ may have further influenced this association. At the same time, university students frequently report high levels of stress and psychological distress.^17^ While such distress does not necessarily indicate a mental disorder, it is an important predictor of later mental health symptoms and disorders. Recognising this distinction is important both to avoid over-pathologising distress and to acknowledge its role as a key confounder in the association between financial strain and depression.

Consequently, there is a need for updated large-scale longitudinal studies that utilise diagnostic criteria and account for prior symptom levels to better understand the role of financial strain in student mental health. The present study addresses this gap by examining multiple dimensions of financial strain as predictors of clinically defined depression in a large student population.

To address this gap, the present study uses prospective data from a large, nationally representative cohort of Norwegian higher education students who completed the SHoT2022 survey and were followed up one year later with diagnostic assessments based on the Composite International Diagnostic Interview version 5.0 (CIDI 5.0). Focusing on depression, a leading contributor to global disability among young people,^18^ we examined the predictive role of seven financial indicators: (a) general financial difficulties, (b) inability to cover a hypothetical emergency expense, (c) annual income, (d) housing cost burden, (e) housing ownership, (f) lack of savings and (g) absence of family financial support. By stratifying results by sex and applying a three-step adjustment approach, we aimed to clarify the extent to which financial strain independently contributes to later depression when accounting for demographic and psychological confounders.

## Method

### Study design, participants and setting

This study is based on data from SHoT, a national survey targeting students in higher education in Norway. The SHoT survey has been conducted every four years since 2010, with the most recent wave, SHoT2022, administered between 8 February and 19 April 2022. SHoT2022 collected comprehensive information on mental and somatic health, lifestyle, social relationships and financial stressors. Detailed methodology for SHoT has been described previously.^19^

SHoT2022 was distributed digitally via a web-based questionnaire to all full-time students in Norwegian higher education institutions, including those studying abroad. Invitations were sent via email and SMS, supported by coordinated efforts from student welfare organisations and educational institutions. Of the 169 572 students invited, 59 544 participated (response rate 35.1%). All participants provided informed consent. For the present study, we included a subsample of 53 362 students who were aged 18–35 years, enrolled full-time and had complete data on key financial and mental health measures. PhD students were not invited to SHoT2022, and information on undergraduate versus graduate/postgraduate status was not collected.

**Fig. 1:**
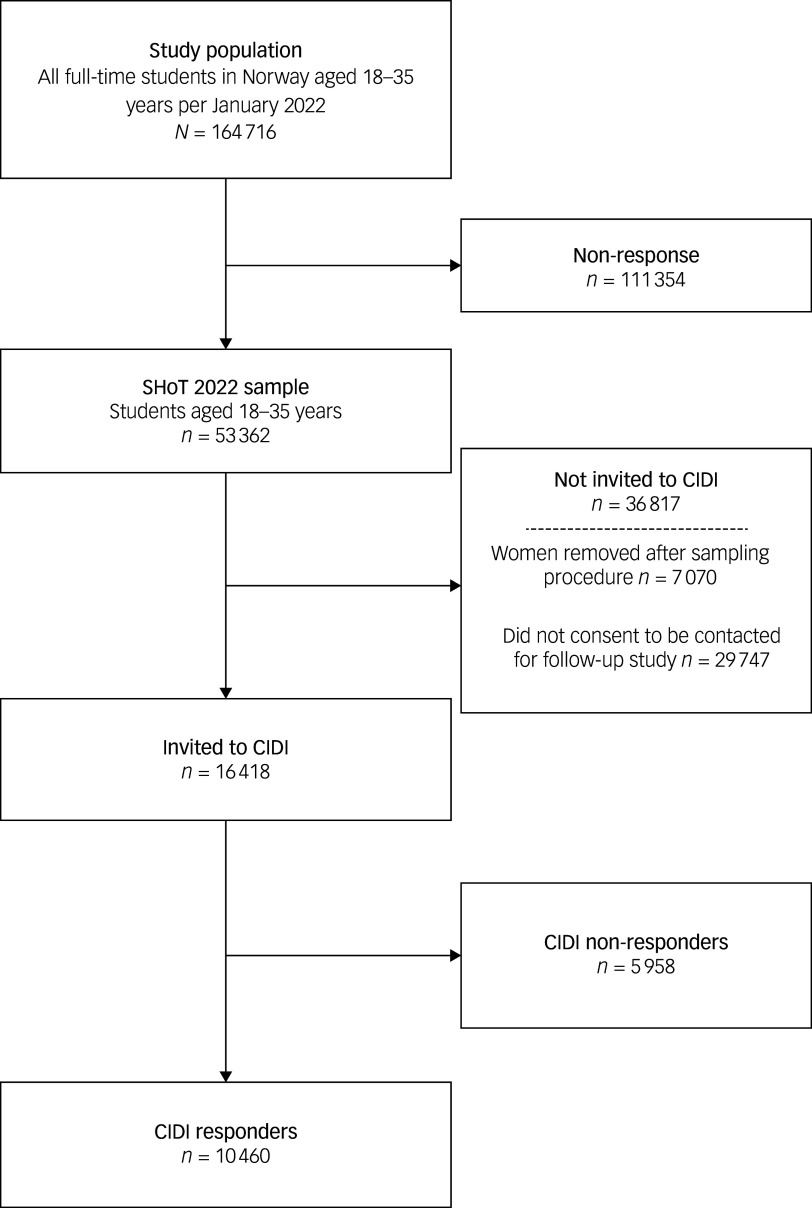
Flowchart of study participants. SHoT, Students’ Health and Wellbeing Study; CIDI, Composite International Diagnostic Interview.

To assess subsequent depression, a follow-up diagnostic study (CIDI study) was conducted approximately one year later, from 24 January to 6 February 2023. Of the 26 311 students who indicated willingness to be recontacted, 16 418 were invited to participate, with oversampling of men to counteract sex imbalance. Ultimately, 10 460 students completed at least one diagnostic module, corresponding to 63.7% of those invited and 17.6% of the baseline SHoT2022 sample. A flowchart of participation is shown in Fig. 1.

### Instruments

#### Sociodemographic information

Age and sex were obtained from the Norwegian national identity number registry. Other background variables were self-reported in SHoT2022, including civil status (single, cohabiting or married), migration background (self or parental birth outside Norway), parental education (categorised in three levels), and whether the respondent had children of their own (student parental status) and accommodation status (dichotomised as living alone versus living with others).

#### Financial indicators

We examined seven self-reported indicators of financial strain, capturing both short- and long-term dimensions of economic vulnerability. General financial difficulties were assessed by the item: ‘In the past 12 months, have you/your household had difficulty covering regular expenses such as food, housing or transport?’ with four response options (‘Never’, ‘Rarely’, ‘Sometimes’, ‘Often’). This measure reflects perceived challenges in meeting everyday costs. An emergency bill was used as an indicator of short-term financial resilience. Respondents were asked whether their household would have been able to cover an unexpected 5000 NOK expense (approximately €450 / $500 USD at the time of data collection). Responses were dichotomised into ‘Able’ versus ‘Unable’. Personal annual income from work (before taxes and deductions, excluding loans and stipends) was reported using multiple brackets. For analysis, these were collapsed into four categories: <10 000 NOK, 10 000–49 999 NOK, 50 000–99 999 NOK and ≥100 000 NOK. These categories correspond approximately to <€900 / <$1000; €900–€4500 / $1000–$4500; €4500–€9000 / $4500–$9000; and ≥€9000 / ≥$9000, respectively. Housing cost burden was self-reported as the proportion of total personal income spent on housing, reported in 10% increments (e.g. 10–19%, 20–29%, etc.). For analysis, categories were collapsed into five groups: 1–19%, 20–39%, 40–59%, 60–79% and 80–100%. Housing ownership was assessed with the item: ‘Who owns the residence you currently live in?’ Response options included: (a) Student welfare organisation, (b) Professional private landlord, (c) Other private landlord, (d) My parents/relatives, (e) I/we own the residence and (f) Other. For analysis, this variable was dichotomised to distinguish between those who owned their residence (I/we own the residence), and those who did not (all other options). Responses indicating ‘Other’ were set to missing due to interpretational ambiguity. Savings behaviour was assessed with the question: ‘Do you set aside money for savings?’ (Yes / No), representing a marker of financial security and planning. Parental financial support was assessed with the item: ‘Do you receive financial help from your family?’ (Yes / No), capturing potential external support buffering economic hardship.

#### Psychological distress: HSCL-25

Symptoms of anxiety and depression at baseline (SHoT2022) were assessed using the 25-item version of the Hopkins Symptom Checklist (HSCL-25), a widely used screening instrument in population studies.^20^ Each item is rated on a four-point Likert scale (1 = ‘not at all’ to 4 = ‘extremely’), and the mean of all items is used to calculate an overall distress score. For this study, psychological distress was dichotomised based on sex-specific cut-offs recently validated in a Norwegian student population: ≥1.96 for men and ≥2.2 for women, which have demonstrated improved balance between sensitivity and specificity compared to the traditional threshold of 1.75.^21^ This binary variable was included in fully adjusted models (Model 3) to account for baseline symptom levels that may confound the relationship between financial stress and subsequent depression.

#### Major depressive episode (MDE): CIDI

A current major depressive episode (MDE) was assessed using the Norwegian version of CIDI 5.0, a self-administered, web-based diagnostic tool developed for the WHO World Mental Health Surveys.^22^ The CIDI assesses 30-day, 12-month and lifetime prevalence of mental disorders according to DSM-5 criteria.^23^ CIDI 5.0 has shown good concordance with clinician-administered diagnostic interviews, the Structured Clinical Interview for DSM-IV (SCID)^24^ and Schedules for Clinical Assessment in Neuropsychiatry (SCAN).^25^ The Norwegian version followed the official translation process.^26^ For the present study, only current depression was included. We defined current MDE as present if reported in the 30 days prior to the CIDI study and excluded individuals with only past (12-month or lifetime) MDE (*n* = 2789), to ensure temporal ordering relative to financial indicators reported in SHoT2022.

### Statistical analyses

All descriptive analyses (Tables 1 and 2) were conducted on unweighted data, in line with reporting conventions for sample characteristics.^27,28^ To address the possibility of bias due to differential response, all regression analyses applied inverse probability weights (IPW).^29^ Such bias may arise if participation in the CIDI follow-up is systematically related to sociodemographic or clinical characteristics. Response probabilities were estimated from a logistic model including baseline sociodemographic variables and HSCL-25, but excluding the seven financial indicators (exposures) to avoid overadjustment in the weighting model. Each participant was weighted by the inverse of their predicted probability of response, ensuring that the weighted analytic sample more closely resembled the full SHoT2022 population.

**Table 1:** Sociodemographic and clinical characteristics in 2022 of the Composite International Diagnostic interview (CIDI) responders, CIDI non-responders and the overall Students’ Health and Wellbeing Study 2022 (SHoT2022) sample

Characteristic	CIDI responders (*n* = 10 460)	CIDI non-responders (*n* = 5958)	*p*-value	SHoT2022^a^(*n* = 53 362)	*p*-value^b^
Age, mean (s.d.)	24.03 (3.28)	23.97 (3.24)	0.24	23.98 (1.85)	0.14
Sex,% (*n*)			0.97		<0.001
Women	70.6 (7386)	70.4 (4196)		66.4 (35 423)	
Men	29.4 (3074)	29.6 (1762)		33.6 (17 939)	
Marital status,% (*n*)			0.16		0.70
Single	51.2 (5359)	50.3 (2994)		51.0 (27 197)	
Boy-/girlfriend	22.4 (2343)	23.7 (1414)		22.8 (12 152)	
Cohabitant	22.7 (2375)	22.5 (1340)		22.6 (12 058)	
Married/registered partner	3.3 (345)	3.0 (178)		3.1 (1667)	
Self and/or parent(s) born abroad,% (*n*)			0.17		0.34
Born in Norway	81.2 (8491)	80.4 (4792)		80.1 (43 052)	
Born outside Norway	10.0 (1043)	10.9 (651)		10.4 (5541)	
Maternal education,% (*n*)			0.04		0.50
Primary	4.4 (457)	5.3 (313)		4.5 (2407)	
Secondary	27.3 (2857)	27.6 (1646)		27.6 (14 707)	
College/university	65.6 (6858)	64.2 (3827)		64.3 (34 326)	
Paternal education,% (*n*)			0.02		0.52
Primary	5.7 (599)	6.8 (406)		6.0 (3182)	
Secondary	35.2 (3678)	24.9 (2078)		35.1 (18 735)	
College/university	54.2 (5674)	52.8 (3145)		53.3 (28 446)	
Financial difficulties,% (*n*)			0.004		0.14
Often	5.8 (609)	8.0 (475)		6.1 (3245)	
Sometimes	18.6 (1941)	20.3 (1206)		18.5 (9898)	
Rarely	20.1 (2106)	20.3 (1207)		20.8 (11 111)	
Never	55.1 (5766)	51.1 (3046)		54.0 (28 801)	
* Missing*	*0.4 (38)*	*0.4 (24)*		*0.6 (307)*	
HSCL-25, Mean (s.d.)	1.88 (0.61)	1.89 (0.61)	0.30	1.86 (0.59)	0.01
* Missing,% (n)*	*0.2 (17)*	*0.3 (24)*		*0.4 (214)*	

HSCL-25, Hopkins Symptoms Checklist – 25 items version.

a. Grand mean for the SHoT2022 sample aged 18–35.

b. Compared with the CIDI responders group (*p*-values based on chi-squared test (categorical variables) or *t*-test (continuous variables).

**Table 2:** Distribution of financial strain indicators among female and male students in the total Students’ Health and Wellbeing Study 2022 (SHoT2022) sample. Percentages and unweighted counts (*n*) are presented for each response option. Pearson chi-square tests were used to assess sex differences

Characteristic	Women, % (*n*)	Men, % (*n*)	Total, % (*n*)	Statistics
Financial difficulty (12 months)				χ^2^ = 196.853, d.f. = 3, *p* < 0.001
Often	6.1 (2412)	5.6 (1116)	6.0 (3528)	
Sometimes	19.9 (7821)	15.8 (3140)	18.5 (10 961)	
Rarely	20.9 (8242)	20.0 (3968)	20.6 (12 210)	
Never	53.1 (20 903)	58.5 (11 600)	54.9 (32 503)	
Could cover NOK 5000 bill				χ^2^ = 110.331, d.f. = 1, *p* < 0.001
No	28.7 (11 249)	24.6 (4867)	27.4 (16 116)	
Yes	71.3 (27 913)	75.4 (14 887)	72.6 (42 800)	
Income (4 categories)^a^				χ^2^ = 41.497, df = 3, *p* < 0.001
<10k NOK	3.2 (1049)	3.6 (567)	3.4 (1616)	
10–50k NOK	19.9 (6414)	19.2 (3011)	19.6 (9425)	
51–100k NOK	30.2 (9764)	27.9 (4374)	29.5 (14 138)	
>100k NOK	46.7 (15 064)	49.3 (7726)	47.5 (22 790)	
Housing cost share (recoded)				χ^2^ = 168.751, d.f. = 4, *p* < 0.001
80–100%	8.2 (3099)	5.3 (1019)	7.3 (4118)	
60–79%	28.1 (10 572)	28.1 (5398)	28.1 (15 970)	
40–59%	30.6 (11 521)	31.6 (6,067)	31.0 (17 588)	
20–39%	21.8 (8188)	22.9 (4397)	22.2 (12 585)	
1–19%	11.2 (4223)	12.0 (2306)	11.5 (6529)	
Housing ownership				χ^2^ = 100.278, df = 1, *p* < 0.001
Own residence	25.0 (7921)	21.5 (5881)	23.4 (13 802)	
Not owner (e.g. rental, student housing, parents, etc.)	75.0 (23 730)	78.5 (21 441)	76.6 (45 171)	
Saves money				χ^2^ = 480.945, d.f. = 1, *p* < 0.001
No	18.3 (7231)	26.1 (5179)	21.0 (12 410)	
Yes	81.7 (32 177)	73.9 (14 649)	79.0 (46 826)	
Receives family help				χ^2^ = 15.458, d.f. = 1, *p* < 0.001
No	58.9 (23 132)	57.2 (11 313)	58.3 (34 445)	
Yes	41.1 (16 148)	42.8 (8465)	41.7 (24 613)	

a. All income values are reported in Norwegian Kroner (NOK).

Associations between financial indicators and subsequent MDEs were assessed using Poisson regression with robust standard errors, yielding risk ratios with 95% confidence intervals (CI). A log link function was applied, ensuring that the models estimated relative risks rather than odds ratios, consistent with Zou’s modified Poisson approach for binary outcomes.^30^ Given the use of IPWs to address differential response, combined with adjustment for baseline covariates (including HSCL-25), the analytic approach can be considered doubly robust, producing consistent estimates if either the response model or the outcome model is correctly specified.^31^

Tests for interaction between sex and financial indicators were non-significant (all ps > 0.05). Regression models were therefore estimated in the full sample, with sex included as a covariate and incorporated into the weighting procedure. To account for confounding and clarify causal direction, a three-step adjustment strategy was applied: (a) unadjusted models, (b) models adjusted for sociodemographic covariates (age, sex, immigrant background, parental education, student parental responsibility, and relationship and accommodation status) and (c) models additionally adjusted for baseline psychological distress (HSCL-25). This final step is particularly important for causal interpretation, as it accounts for pre-existing symptom burden at baseline. By including this measure, we reduce the risk that the observed associations merely reflect reverse causation (e.g. underlying distress leading to both financial strain and a later MDE). Instead, the fully adjusted models provide a clearer indication of the independent contribution of financial strain to subsequent depression, above and beyond prior mental health status.

Given that seven financial indicators were examined, p-values from the fully adjusted models were corrected for multiple testing using the false discovery rate (FDR)^32^ procedure to reduce the risk of type I error. While the indicators are conceptually related and expected to be correlated, we did not formally test or combine them, as the aim was to evaluate their independent associations with depression. All analyses were performed using IBM SPSS Statistics version 30 for Windows.^33^

### Ethics

The study follows the STROBE reporting guidelines. Ethical approval for SHoT 2022 was granted by the Regional Committee for Medical and Health Research Ethics in Western Norway (reference no. 326437). All participants gave electronic informed consent after receiving comprehensive information about the study procedures.

## Results

### Sample characteristics and representativeness

Table 1 presents key sociodemographic and clinical characteristics of participants in the CIDI follow-up study compared to the full SHoT2022 sample. CIDI responders were slightly older and more likely to be women, with a higher proportion reporting financial difficulties. The demographic profile was otherwise largely comparable to the full SHoT2022 sample. Baseline psychological distress scores (HSCL-25) were marginally lower among responders than non-responders (Cohen’s *d* = 0.03), indicating minimal response bias. See Table 1 for detailed comparisons.

### Financial indicators

In the total sample of students, over half (54.9%) reported no financial difficulties in the past 12 months, while 6.0% reported often struggling to meet basic expenses such as food, housing and transport. More than one in four students (27.4%) stated that they would not be able to cover an unexpected NOK 5000 (approximately €450 / $500) expense. Although nearly half (47.5%) reported an annual income of at least NOK 100 000 (≥€9000 / ≥$9000), a substantial proportion had modest earnings, with 22.9% reporting income below NOK 50 000 (<€4500 / <$4500). Housing costs represented a major financial burden for many students, with 35.4% spending 60% or more of their income on housing. Most students (79.0%) reported setting aside money for savings, and 41.7% received financial help from family.

Significant sex differences emerged across nearly all indicators. Women were more likely than men to report financial difficulties, with 53.1% of women reporting no problems compared to 58.5% of men (*p* < 0.001). A larger proportion of women also reported being unable to cover an unexpected bill (28.7 *v*. 24.6%, *p* < 0.001). Although the overall income distribution was similar, slightly more men reported earnings in the highest income bracket (49.3 *v*. 46.7%, *p* < 0.001). The burden of housing costs was more pronounced among women, with 37.5% spending at least 60% of their income on housing, compared to 33.4% of men (*p* < 0.001). Women were also more likely to report saving money regularly (81.7 *v*. 73.9%, *p* < 0.001), whereas financial support from family was slightly more common among men (42.8 *v*. 41.1%, *p* < 0.001).

In the weighted CIDI sample, most financial strain indicators were associated with an elevated risk of an MDE one year later. As shown in Table 3, the strongest associations were observed for self-reported financial difficulty, inability to cover an emergency bill and lack of savings. Adjusting for sociodemographic variables (Model 2) had little impact on estimates, while further adjustment for baseline psychological distress (Model 3) led to substantial attenuation. Several associations remained statistically significant after full adjustment, whereas others became non-significant, suggesting partial confounding by existing psychological distress.

**Table 3:** Estimated prevalence and relative risk of depression (reference = lowest risk group), by financial strain indicator

	MDE% (IPW-weighted, adj. for age and sex)	Relative risk (Model 1)	Relative risk (Model 2)	Relative risk (Model 3)	FDR q-value (Model 3)
Financial difficulty (12 months)					0.002
Often	45.4	3.55 (2.97–4.22)	3.48 (2.91–4.14)	1.53 (1.28–1.83)	
Sometimes	32.9	2.57 (2.26–2.92)	2.52 (2.21–2.86)	1.43 (1.25–1.63)	
Rarely	23.1	1.81 (1.57–2.07)	1.78 (1.55–2.05)	1.32 (1.14–1.52)	
Never	12.8	1.00 (ref)	1.00 (ref)	1.00 (ref)	
Could cover NOK 5000 bill					0.002
No	31.9	2.02 (1.82–2.24)	1.96 (1.76–2.18)	1.27 (1.14–1.41)	
Yes	15.8	1.00 (ref)	1.00 (ref)	1.00 (ref)	
Income (4 categories)					0.132
<10k NOK	24.8	1.45 (1.08–1.93)	1.44 (1.06–1.91)	1.21 (0.89–1.60)	
10–50k NOK	19.0	1.12 (0.95–1.31)	1.12 (0.95–1.31)	1.00 (0.85–1.17)	
51–100k NOK	16.5	0.97 (0.84–1.13)	0.99 (0.85–1.14)	0.92 (0.79–1.06)	
>100k NOK	17.1	1.00 (ref)	1.00 (ref)	1.00 (ref)	
Housing cost share					0.303
80–100%	26.9	1.46 (1.16–1.86)	1.50 (1.19–1.90)	0.99 (0.78–1.25)	
60–79%	22.1	1.20 (1.00–1.46)	1.25 (1.04–1.52)	1.06 (0.88–1.29)	
40–59%	19.7	1.07 (0.89–1.30)	1.12 (0.93–1.36)	1.03 (0.85–1.24)	
20–39%	15.6	0.85 (0.69–1.05)	0.88 (0.72–1.10)	0.93 (0.75–1.15)	
10–19%	18.4	1.00 (ref)	1.00 (ref)	1.00 (ref)	
Housing ownership					0.026
Not owner	21.3	1.49 (1.27–1.76)	1.41 (1.20-1.66)	1.16 (0.90-1.36)	
Own residence	14.1	1.00 (ref)			
Saves money					0.002
No	30.4	1.71 (1.52–1.92)	1.67 (1.48–1.87)	1.23 (1.09–1.38)	
Yes	17.8	1.00 (ref)	1.00 (ref)	1.00 (ref)	
Receives family help					0.231
No	19.3	0.92 (0.83–1.02)	0.90 (0.81–1.00)	0.95 (0.85–1.06)	
Yes	21.5	1.00 (ref)	1.00 (ref)	1.00 (ref)	

Relative risks and 95% confidence intervals for a major depressive episode (MDE) one year later according to financial indicators. Model 1: unadjusted. Model 2: adjusted for sociodemographic covariates. Model 3: additionally adjusted for baseline psychological distress (HSCL-25). Given that seven financial indicators were examined, *p*-values in Model 3 were corrected for multiple testing using the false discovery rate (FDR) procedure; corresponding *q*-values are reported. NOK, Norwegian Kroner.

A clear dose–response relationship was found for financial difficulty. Compared to those reporting no difficulty, those who reported difficulties often had a risk ratio of 3.55 (95% CI: 2.97–4.22) in Model 1, which remained stable in Model 2 (risk ratio 3.48; 2.91–4.14) but was attenuated to 1.53 (1.28–1.83) in Model 3. Those reporting difficulty ’sometimes’ or ‘rarely’ had adjusted risk ratios of 1.43 (1.25–1.63) and 1.32 (1.14–1.52), respectively, in Model 3, indicating a persistent, graded association even after accounting for baseline mental health.

Participants unable to cover an unexpected 5000 NOK bill showed a significantly increased MDE risk in all models: risk ratio 2.02 (1.82–2.24) in Model 1, risk ratio 1.96 (1.76–2.18) in Model 2 and risk ratio 1.27 (1.14–1.41) after full adjustment. Similarly, not having savings was associated with higher MDE risk (risk ratio 1.71 in Model 1; 1.52–1.92), with the effect remaining significant in Model 3 (risk ratio1.23; 1.09–1.38). For housing cost burden, spending 80–100% of income on housing was associated with increased risk in Models 1 and 2 (risk ratio 1.46 and 1.50, respectively), but attenuated to 0.99 (0.78–1.25) in Model 3. Similar attenuation to non-significance was observed for other housing burden categories, indicating these associations may be explained by underlying psychological distress. For housing ownership, participants who did not own their residence had a significantly higher risk of MDE in both Model 1 (risk ratio 1.49; 95% CI: 1.27–1.76) and Model 2 (risk ratio 1.41; 1.20–1.66). However, the association was attenuated and no longer statistically significant in Model 3 (risk ratio 1.16; 0.90–1.36). Regarding income, individuals with annual earnings below 10 000 NOK had a significantly elevated MDE risk in Models 1 and 2 (risk ratio 1.45; 1.08–1.93 and risk ratio 1.44; 1.06–1.91, respectively), but this association was no longer statistically significant in Model 3 (risk ratio 1.21; 0.89–1.60). No significant income gradient was observed across other income brackets. Finally, receiving financial support from family showed no association with MDE risk in any model.

## Discussion

In this large, prospective study of Norwegian higher education students, we found that frequent general financial difficulties were most consistently associated with increased risk of an MDE one year later, even after adjustment for sociodemographic factors and baseline psychological distress. Associations for other financial indicators, including inability to cover an emergency bill, low annual income, housing cost burden, housing ownership and lack of savings, were generally weaker and in several cases attenuated after adjustment for baseline distress. These findings suggest that while multiple aspects of financial strain may be relevant, persistent financial difficulties appear to represent the most robust risk factor.

Among the indicators examined, frequent general financial difficulties showed the strongest and most consistent associations with later depression. Associations for emergency expense inability and lack of savings were also observed, though more modest and less stable after adjustment. This aligns with theoretical models emphasising the role of subjective financial strain in shaping psychological outcomes.^10,11^ The clear dose–response pattern, particularly in unadjusted and sociodemographically adjusted models, is in line with a cumulative stress framework, where increasing financial pressure gradually undermines coping capacity and resilience.

The attenuation in fully adjusted models suggests that for some, the association between financial hardship and depression remains stable over time, especially among those experiencing frequent financial hardship. This attenuation underscores the importance of baseline distress, but also highlights that we cannot rule out reverse causality, as pre-existing depression may have influenced both financial strain and a subsequent MDE. A bidirectional influence may also be present, whereby depressive symptoms reduce the likelihood of engaging in part-time work and thereby further contribute to financial hardship.^34^

Income-related findings were less consistent. Only men in the lowest income category showed a statistically significant increased risk of depression, echoing earlier findings that economic insecurity may affect mental health differently by sex.^35^ The interaction terms were non-significant and patterns should be interpreted with caution and may warrant further investigation. While the general lack of association between income and depression contrasts with findings from the general adult population, this relationship may be harder to detect in student samples.^36,37^ Students’ financial situations are often shaped by factors beyond earned income, such as financial support from parents or access to student loans. Moreover, a given income level may not correspond to the same cost of living across different regions. As such, students’ ability to cover their expenses may be better reflected by the alternative financial measures used in this study. Echoing findings by others,^38^ low income may only become problematic when it fails to cover essential expenses, at which point it may be more accurately captured by the two other measures: emergency expense capacity and financial strain.

The predictive value of the emergency expense item, a proxy for financial buffering, further highlights the salience of short-term material insecurity. Previous work suggests that a lack of financial margin, even in the absence of poverty, can heighten distress through increased exposure to daily stressors and reduced perceived control.^39^ Similarly, not saving money was independently associated with increased MDE risk, supporting the relevance of future-oriented financial behaviour as a marker of both economic and psychological vulnerability. However, these associations weakened after full adjustment, suggesting that they may be secondary to baseline distress. Housing cost burden also showed elevated risk in less adjusted models, but associations were attenuated after controlling for baseline distress. Receiving financial help from family was not associated with depression risk, possibly reflecting the limited protective effect of such support when economic strain persists despite receiving assistance.

Together, our findings suggest that persistent financial difficulties are the most salient financial risk factor for student mental health, with other indicators providing complementary but less robust evidence. The limited attenuation between the models suggests that parental education, relationship status and migration background only partially account for the financial-depression link, possibly due to the relatively homogeneous socioeconomic context of Norwegian higher education. However, as data on parental income were not available, we relied on parental education as a proxy for socioeconomic position, which may not fully capture family financial circumstances.

These findings are consistent with previous cross-sectional research linking financial difficulties to depression and anxiety among university students.^9–12,14^ However, by leveraging longitudinal data and diagnostic outcomes based on the CIDI 5.0, our study adds important evidence of temporal ordering and diagnostic validity. In doing so, it overcomes several limitations of prior work, which has often relied on self-reported symptom scales or cross-sectional designs.^15^ The persistent associations observed even after adjusting for baseline distress suggest that financial stressors may contribute to or exacerbate depressive symptoms over time, rather than simply co-occurring with them.

The temporal design also raises important interpretive questions. Specifically, the lack of repeated measurement points makes it difficult to determine whether the observed associations reflect transient difficulties tied to the student phase of life, or more enduring vulnerabilities. It is plausible that mental health challenges may diminish as students transition into the workforce and achieve greater financial stability. If so, these findings could align with broader literature highlighting the long-term health and socioeconomic benefits of higher education.^40^ Nevertheless, the burden of distress during the study period remains substantial and warrants policy and institutional attention, regardless of long-term outcomes.

The findings carry several implications for policy and practice. While causality cannot be established, and bidirectional effects remain possible, the consistent associations observed between financial strain and subsequent depression highlight that financial stressors should at least be considered when designing efforts to support student mental health. Despite relatively generous public financial support, many Norwegian students report difficulties meeting basic expenses^14^ suggesting that current systems may not fully buffer against economic stress. We cannot conclude that providing additional financial resources would directly reduce depression, but it is plausible that measures aimed at alleviating students’ financial strain could indirectly contribute to improved well-being. Policymakers could therefore consider reviewing the adequacy and structure of existing student support mechanisms, such as grants or targeted subsidies, as one potential strategy to reduce financial vulnerability. Structural changes, such as assuring access to affordable student housing as well as reasonably priced services such as cafeterias and sports facilities could also positively impact students’ financial well-being. In university health services, financial stressors could be acknowledged as relevant contextual factors when assessing mental health risk. Finally, offering access to voluntary financial counselling or guidance on budgeting and resource navigation may provide additional support for students experiencing financial strain, and could be explored as a low-cost supplement to existing well-being services. Norway represents a particularly relevant context for these issues: while being a high-income welfare state with free tuition and relatively generous student loans, the cost of living is among the highest in Europe. Students are estimated to need at least NOK 11 000–13 700 per month (€1100–1300) to cover essentials such as housing, food and transport.^41^ Alcohol, central to student social life, is also particularly expensive; hazardous use remains common and is linked to lower life satisfaction and psychological distress.^42^ Together, these factors may exacerbate both financial strain and mental health vulnerability.

This study has several strengths, including its large, national sample, longitudinal design, use of multiple financial indicators, sex-weighted analyses and use of a validated diagnostic tool (CIDI 5.0) to assess depression. Adjustment for baseline psychological distress enhances interpretability by isolating the independent contribution of financial strain. However, some limitations warrant mention. First, while the prospective design allows for temporal ordering, causality cannot be definitively inferred. Reverse causality, where pre-existing mental health problems lead to financial difficulties, could also account for part of the association. Although individuals with past 12-month or lifetime MDEs were excluded in the CIDI follow-up to ensure temporal ordering, we could not exclude participants with current depression at baseline. Instead, we adjusted for baseline psychological distress (HSCL-25), which captures symptoms but is not equivalent to a diagnostic assessment. Second, response rates were modest for both baseline (35%) and follow-up (64% conditional response), and participation bias, especially under-representation of those with severe distress, cannot be ruled out. Third, baseline mental health was assessed with the HSCL-25, which captures symptoms of distress but is not the typical gold-standard screening instrument used in epidemiological studies (e.g.the Patient Health Questionnaire-9 for depression or Generalised Anxiety Disorder-7 for anxiety). Fourth, although CIDI 5.0 is validated, it remains a self-administered tool; future studies should consider clinical validation in student populations. Finally, although we included a broad range of financial indicators, some (e.g. home ownership, savings, modest earnings) may be less applicable to most undergraduate students. Moreover, our savings measure did not distinguish between those unable versus those unwilling to save, potentially conflating financial strain with financial behaviour.

In conclusion, this study shows that persistent financial difficulties, in particular, are a significant risk factor for later depression among higher education students, even in a high-income welfare state like Norway. The results underscore that free tuition and basic financial support may not be sufficient to buffer all students from economic stress and its mental health consequences. As rates of mental distress rise globally among young adults, addressing financial vulnerability should be considered a key component of public health and university-based mental health strategies.

## Data Availability

Data sharing is restricted by Norwegian data protection regulations and GDPR. However, researchers can request access to participant data by contacting the SHoT publication committee (borge.sivertsen@fhi.no). Data access requires approval from the Norwegian Regional Committee for Medical and Health Research Ethics (https://helseforskning.etikkom.no). The data-set is managed by the NIPH, and further information regarding data access procedures is available at https://www.fhi.no/en/more/access-to-data.

## Analytic code availability

The analytic code supporting the reported findings is available upon request from the corresponding author, B.S.
